# Optimization and construct validity of approaches to preclinical grip strength testing

**DOI:** 10.1002/jcsm.13300

**Published:** 2023-08-13

**Authors:** Gregory Owendoff, Alissa Ray, Prameela Bobbili, Leatha Clark, Cory W. Baumann, Brian C. Clark, W. David Arnold

**Affiliations:** ^1^ Department of Neurology The Ohio State University Wexner Medical Center Columbus OH USA; ^2^ Ohio Musculoskeletal and Neurological Institute (OMNI) Ohio University Athens OH USA; ^3^ Department of Biomedical Sciences Ohio University Athens OH USA; ^4^ Department of Family Medicine Ohio University Athens OH USA; ^5^ Department of Physical Medicine and Rehabilitation The Ohio State University Wexner Medical Center Columbus OH USA; ^6^ Department of Neuroscience The Ohio State University Wexner Medical Center Columbus OH USA; ^7^ Department of Physiology and Cell Biology The Ohio State University Wexner Medical Center Columbus OH USA; ^8^ NextGen Precision Health University of Missouri Columbia MO USA; ^9^ Department of Physical Medicine and Rehabilitation University of Missouri Columbia MO USA; ^10^ Department of Neurology University of Missouri Columbia MO USA; ^11^ Department of Medical Pharmacology and Physiology University of Missouri Columbia MO USA

## Background

Grip strength is a robust biomarker showing good reliability^1^, ^2^ and prediction of negative health outcomes.^3^, ^4^ Low grip strength is associated with disability and premature death^5^, ^6^, ^7^, ^8^, ^9^ and is more strongly associated with frailty than chronological age.^10^ Accordingly, recent updates to consensus definitions of sarcopenia focus on low grip strength as the primary characteristic as opposed to low muscle mass.^11^, ^12^ Because rodent models are indispensable tools in aging research, scientists have reverse‐translated grip testing as a key outcome in the context of sarcopenia.^13^, ^14^, ^15^, ^16^ Serendipitous development of preclinical grip testing has resulted in a variety of protocols that have not been extensively examined and compared.^13^, ^14^, ^15^, ^17^ Variability, due to motivation, temperament, and other factors such as pain, is inherent in preclinical behavioural assessments.^18^ Limited research has focused on standardizing preclinical grip testing, validation of methods against other functional measures, and investigating how preclinical grip data compare to data from humans and whether these tests even measure the same construct. Additionally, prior work has not examined between‐day reliability of grip testing in rodents. This work was undertaken to inform rigorous preclinical grip testing.

## Methods

### Preclinical grip study

**Figure 1:**
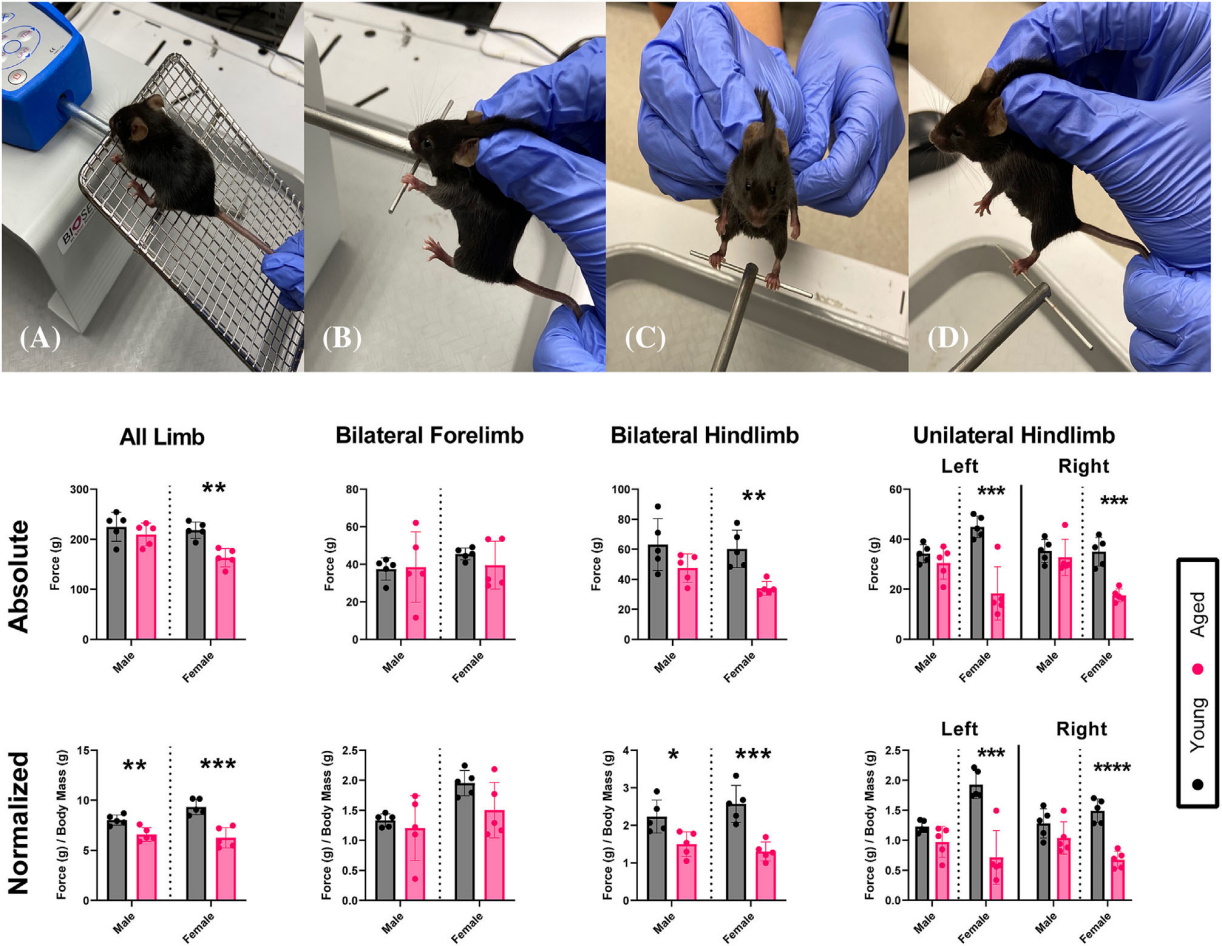
Preclinical grip strength testing. Top row: Grip strength techniques: (A) all limb, (B) bilateral forelimb, (C) bilateral hindlimb, and (D) unilateral hindlimb (left hindlimb shown). Middle row: Absolute grip strength values in young versus aged mice. Bottom row: Grip strength normalized to body mass. Unpaired *t*‐tests compared young versus aged groups: *P < 0.05, **P < 0.01, ***P < 0.001, ****P < 0.0001.

Studies (approved by The Ohio State University Wexner Medical Center) included two age groups of C57BL/6 mice (50% female) (*n* = 10, 6 months, *n* = 10, 24 months, equivalent to 70 human years, roughly equal to the age of our older adult data^19^) maintained on a 12‐h light–dark cycle with free access to food and water. A single rater performed grip strength (reported in grams, g) at 8–10 am in the following order: all limb, bilateral forelimb, bilateral hindlimb, left unilateral hindlimb, and right unilateral hindlimb (BIO‐GS3, Bioseb, Vitrolles, France) (*Figure* *1*). For bilateral forelimb, bilateral hindlimb, and unilateral hindlimb tests, mice were scruffed while maintaining limb range of motion. For bilateral forelimb, bilateral hindlimb, left hindlimb, and right hindlimb tests, mice were held perpendicular to the vector of the force transducer and allowed to grip the attachment and then pulled straight back away from the bar (parallel with the floor), using a consistent speed and force by the experimenter across all trials and replicates. Three trials were averaged and used for analyses. A week later, grip testing was repeated by the same rater, blinded to prior results. Peak plantarflexion muscle contractility torque of the right hindlimb was assessed following stimulation trains at 5, 30, 45, 75, 90, 125, and 150 Hz for 1 s, with 60 s of rest between trains to avoid fatigue, as previously described^20^ (1300A, Aurora Scientific, Aurora, ON, Canada). Mice were euthanized with carbon dioxide inhalation and cervical dislocation. Bilateral gastrocnemius and soleus wet muscle mass were assessed and averaged; the plantaris was included in the gastrocnemius (labelled ‘gastrocnemius’ for simplicity).

### Clinical grip and lean mass outcomes

Grip strength (3 trials, bilaterally; 4 trials if trials differed by >3 kg) and bilateral hand and forearm lean mass data (via dual energy X‐ray absorptiometry, DXA) obtained in the UNCODE Study conducted at Ohio University (which reviewed and approved the human subjects testing protocol; NCT02505529) were analysed. Peak grip strength and lean mass for the bilateral upper limbs were averaged. Procedures and inclusion and exclusion criteria were previously described.^21^, ^22^, ^23^

### Statistical analysis

Analyses was performed using Graph Pad Prism (GraphPad Software Inc., La Jolla, CA, USA), Excel (Microsoft Corp., Redmond WA, USA), and SPSS (IBM Corp., Armonk NY, USA). *P* < 0.05 was considered significant.

## Results

### Preclinical grip testing

#### Grip intra‐rater reliability

All limb grip showed modest reliability with the lowest CV [ICC 0.45 (0.02, 0.74) and CV: 8.6%], and left hindlimb showed the poorest reliability [ICC: 0.10 (95% CI: −0.35, 0.51) and CV: 21.5%]. Otherwise, modest values for ICC and CV were noted for right hindlimb [ICC: 0.41 (−0.03, 0.71) and CV: 14.1%], all limb [ICC; 0.45 (0.02, 0.74) and CV: 8.6%], bilateral forelimb [ICC: 0.53 (0.11, 0.79) and CV: 16.6%], and bilateral hindlimb [ICC: 0.52 (0.11, 0.78) and CV: 14.8%].

#### Grip strength in young versus aged mice

All limb grip was 7% lower in aged (209.5 ± 22.8 g) versus young males (224.9 ± 28.8 g), and 25% lower in aged (163.2 ± 18.3 g) versus young females (218.3 ± 16.4 g) (Figure 1). Bilateral forelimb grip was similar in aged (38.6 ± 18.8 g) versus young males (37.5 ± 5.9 g). Bilateral forelimb grip was 13% lower in aged (39.6 ± 12.8 g) versus young females (45.5 ± 3.1 g). Bilateral hindlimb grip was 25% lower in aged (47.5 ± 9.5 g) versus young males (63.1 ± 17.3 g), and 44% lower in aged (34.0 ± 4.6 g) versus young females (60.3 ± 12.5 g). Left hindlimb grip was 11% lower in aged (30.5 ± 6.5 g) versus young males (34.2 ± 3.6 g), and 59% lower in aged (18.3 ± 10.7 g) versus young females (44.9 ± 4.3 g). Right hindlimb grip was 7% lower in aged (32.8 ± 7.3 g) versus young males (35.3 ± 4.6 g), and 50% lower in aged (17.5 ± 2.6 g) versus young females (35.0 ± 5.8 g). Normalized all limb grip was 18% lower in aged (6.59 ± 0.69 g/g) versus young males (8.03 ± 0.50 g/g), and 33% lower in aged (6.26 ± 1.01 g) versus young females (9.35 ± 0.78 g/g). Normalized bilateral forelimb grip was 10% lower in aged (1.20 ± 0.54 g/g) versus young males (1.34 ± 0.11 g/g), and 23% lower in aged (1.50 ± 0.46 g/g) versus young females (1.95 ± 0.21 g/g). Normalized bilateral hindlimb grip was 33% lower in aged (1.50 ± 0.33 g/g) versus young males (2.23 ± 0.44 g/g). Normalized bilateral forelimb grip was 49% lower in aged (1.31 ± 0.25 g/g) versus young females (2.57 ± 0.49 g/g). Normalized left hindlimb grip was 21% lower in aged (0.97 ± 0.26 g/g) versus young males (1.23 ± 0.10 g/g), and 63% lower in aged (0.72 ± 0.45 g/g) versus young females (1.93 ± 0.23 g/g). Normalized right hindlimb grip was 19% lower in aged males (1.04 ± 0.27 g/g) versus young males (1.28 ± 0.24 g/g), and 54% lower in aged females (0.68 ± 0.14 g/g) versus young females (1.49 ± 0.20 g/g).

#### Grip strength versus muscle contractility and mass

**Figure 2:**
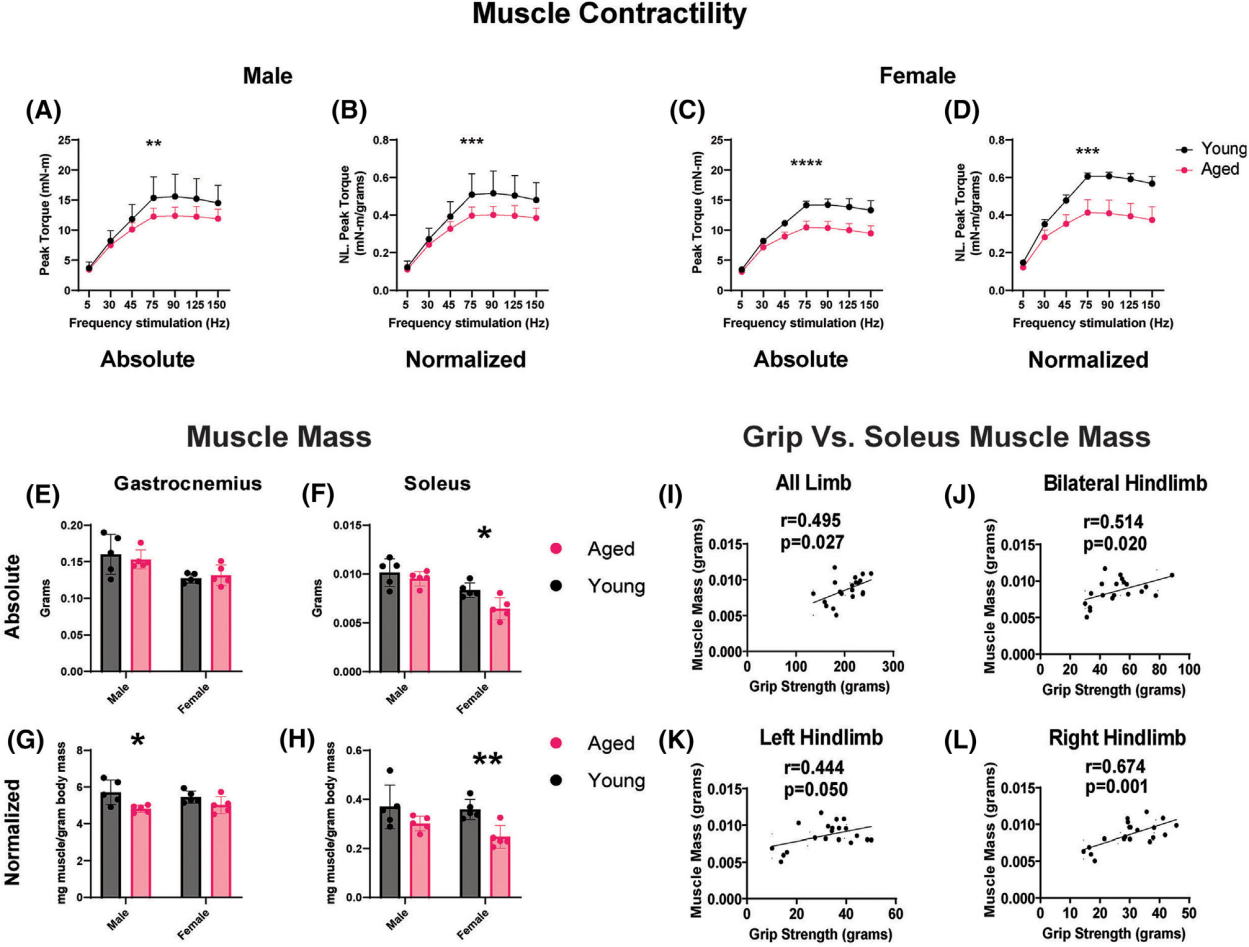
Muscle mass and contractility. (A–D) Plantarflexion muscle contractility. Absolute and normalized plantarflexion torque in aged and young male (A, B) and female (C, D) mice. Each group size was *n* = 5. Repeated measure two‐way ANOVA compared age*stimulation frequency: **P < 0.01, ***P < 0.001, ****P < 0.0001. (E–H) Age‐related differences in muscle mass. Absolute gastrocnemius and soleus mass (E, F) and gastrocnemius and soleus mass normalized to body mass (G, H). Bars graphs indicate mean and standard deviation. Unpaired *t*‐tests compared young versus aged groups: *P < 0.05, **P < 0.01. (I–L) Association between muscle mass and grip strength. Correlation of soleus muscle mass versus grip strength from all limb (I), bilateral hindlimb (J), left hindlimb (K), and right hindlimb (L) tests. Bilateral forelimb grip (data not shown) was not significantly correlated with soleus (*r* = 0.019, *P* = 0.937). Gastrocnemius was not significantly correlated with all limb (*r* = 0.432, *P* = 0.057), bilateral hindlimb (*r* = 0.295, *P* = 0.207), left hindlimb (*r* = −0.078, *P* = 0.743), right hindlimb (*r* = 0.269, *P* = 0.251), or bilateral forelimb grip (*r* = 0.239, *P* = 0.310) muscle mass (data not shown). R, Pearson correlation coefficients.

Muscle contractility demonstrated reduction in aged male and female mice when compared as absolute torque as well as torque normalized to body mass (Figure 2A–D). When examining across stimulation rates (5–150 Hz, twitch to maximum tetanic contractions), significant correlations with all grip techniques were observed, but correlation varied between frequencies and grip approaches (Table 1). Absolute and normalized muscle mass (to body mass) were compared between ages (Figure 2E–H). All limb, bilateral hindlimb, and unilateral hindlimb measures showed significant correlation with muscle mass of the soleus (Figure 2I–L) but not the gastrocnemius (not shown); bilateral forelimb grip was not significantly correlated with soleus or gastrocnemius muscle mass (not shown).

**Table 1:** Correlation of preclinical grip strength and plantarflexion contractility.

Grip Method	5 Hz	30 Hz	45 Hz	75 Hz	90 Hz	125 Hz	150 Hz
**All limb**							
*r*	0.384	0.640*	0.488*	0.488*	0.325	0.019	−0.093
*P* value	0.095	**0.002**	**0.029**	**0.029**	0.162	0.936	0.698
**Bilateral forelimb**							
*r*	0.537*	0.407	0.643*	0.511*	0.504*	0.367	0.331
*P* value	**0.015**	0.075	**0.002**	**0.021**	**0.023**	0.111	0.154
**Bilateral hindlimb**							
*r*	0.299	0.545*	0.352	0.453*	0.394	0.224	0.097
*P* value	0.200	**0.013**	0.128	**0.045**	0.086	0.343	0.686
**Left hindlimb**							
*r*	0.236	0.554*	0.030	0.073	−0.023	−0.146	−0.195
*P* value	0.317	**0.011**	0.900	0.761	0.924	0.539	0.411
**Right hindlimb**							
*r*	0.316	0.527*	0.181	0.244	0.133	−0.0818	−0.147
*P* value	0.175	**0.017**	0.446	0.301	0.576	0.732	0.536

Items in bold indicate statistically significant results (*p* < 0.05).

### Clinical grip testing

**Figure 3:**
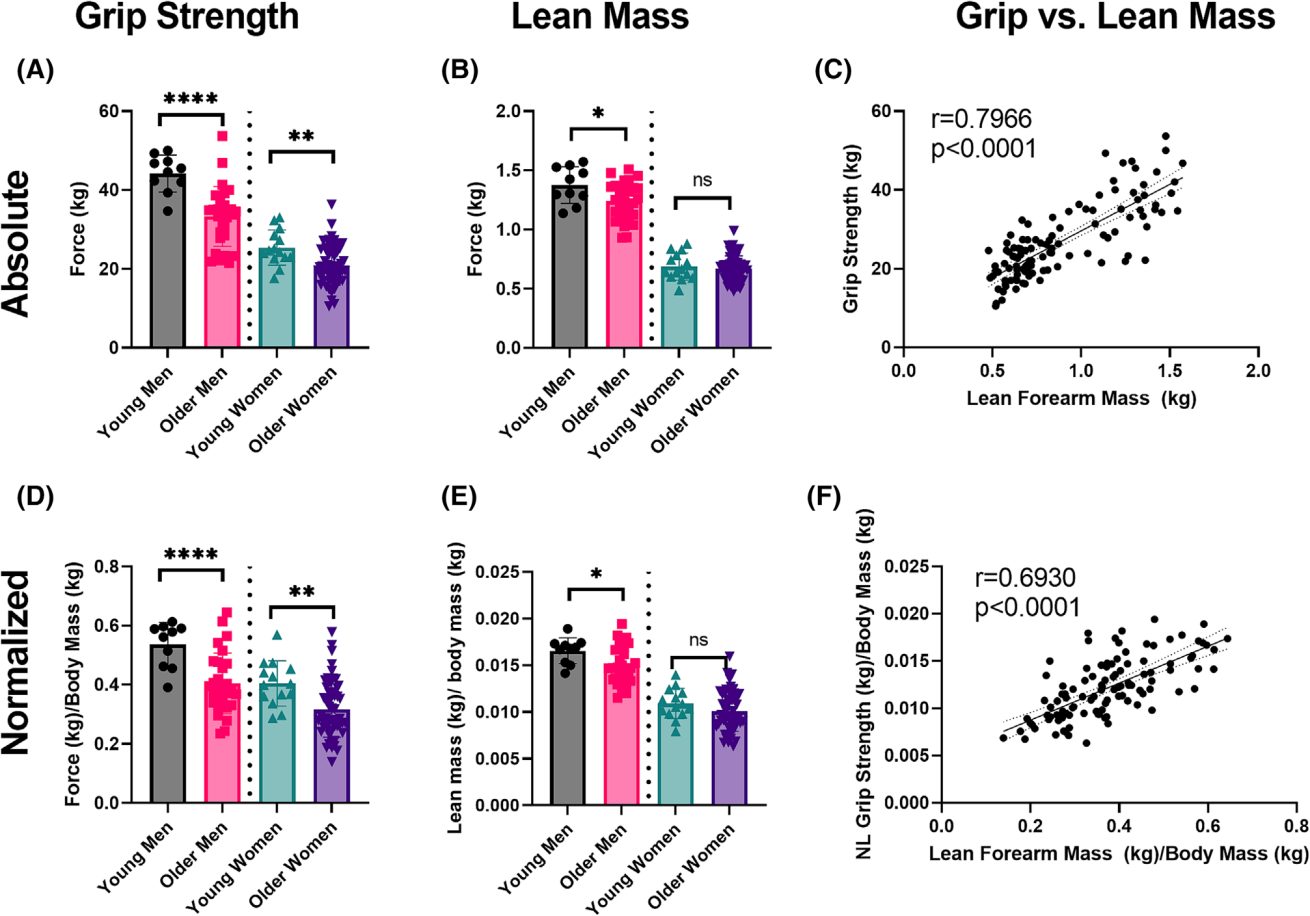
Clinical grip strength and forearm lean mass in young and older adults. Grip strength (A) and forearm lean mass (B) in young and older men and women. Grip and lean mass data were averaged across bilateral upper limbs. (C) Correlation between grip strength and lean mass (Pearson correlation coefficient). Similar results were noted for (D) grip normalized to body mass, (E) forearm lean mass normalized to body mass, and (F) correlations between normalized grip and lean mass. Unpaired *t*‐tests compared young versus older groups: *P < 0.05, **P < 0.01, ****P < 0.0001. R, Pearson correlation coefficient.

Data from 84 older adults [29 men (35%), mean age: 75.5 ± 6.4 years; 55 women (65%), mean age: 74.7 ± 7.0 years] and 24 young adults [10 men (42%), mean age: 22.9 ± 1.4 years; 14 women (58%), mean age: 21.4 ± 1.8 years] were analysed. Grip strength was significantly reduced in older men (−25%) and older women (−18%) compared with younger counterparts (Figure 3A). Lean mass was significantly reduced in older men (−10%) but not women compared with younger counterparts (Figure 3B). Grip strength showed a moderately strong correlation with lean mass (Figure 3C).

## Discussion

Differences between clinical and preclinical grip must be considered when reverse translating methods to mice. Clinical grip testing is volitional whereas preclinical testing depends on reflexive responses. Prior clinical studies have consistently shown ICC ≥ 0.80 for repeated grip strength testing.^1^ No data is available regarding the reliability of grip strength methods in mouse models. One study tested grip strength across three successive trials at a single study timepoint (ICC ranging 0.363–0.803) but did not assess reliability across days.^24^ Our study showed that preclinical grip testing methods are less reliable compared to prior clinical studies. Based on CV, all limb grip testing was the most reliable method; based on ICC, bilateral hindlimb and forelimb grip testing were the most reliable methods. Thus, when choosing a method for grip assessment in aged mice where hindlimb assessment is critical, both all limb and bilateral hindlimb methods appear to be the best options for repeatability. Of note, how mice are grasped, tail or scruffing, was not assessed herein, but might impact results. Thus, further work is needed to better refine preclinical grip testing protocols.

The relationships between grip strength and indices of muscle mass were explored in a clinical cohort to compare these same relationships in mice. The age‐related differences in grip strength noted in both our clinical and preclinical age comparisons were more overt as compared with losses of lean/muscle mass. These findings are aligned with the continued evolution of sarcopenia diagnostic criteria, which are increasingly focused on loss of muscle function rather than size/mass.^3^, ^11^, ^12^ We found moderately strong relationships between clinical grip strength and DXA estimates of lean mass in humans. The muscles that are recruited during grip testing in mice have not been determined, and thus, we used soleus and gastrocnemius muscle mass as proxy measures of muscle mass. Associations in mice were considerably less robust than in the clinical cohort. However, for select grip methods, associations were comparable. Here, it is important to point out the possibility that large differences in sample sizes and size of mouse muscles versus lean mass in humans could impact correlations.

Our work provides insight into the construct validity of grip testing in rodents, which shows reasonable overlap with the conceptual elements of clinical grip testing. In humans, grip is a highly evolved, complex task that imposes great demands on the central nervous system.^25^ Tests of maximum grip strength require that the human brain modulates recruitment of 19 muscles within the hand and another 20 muscles located in the forearm in a spatially and temporally differentiated pattern. Thus, the force measured during grip strength testing depends not only on activation of muscles that flex the fingers but also on the ability of the nervous system to engage muscles that orient the fingers as well as those that stabilize the hand and wrist. Impairments in the nervous system to fully activate the grip musculature become exacerbated with advancing age (for a review, see Clark and Carson^26^). Neural impairment likely explains why ~60% of variability in grip strength was explained by muscle mass in humans. We speculate that in mice, the even lower association between grip strength and muscle mass is due to similar neural impairments but that broader issues related to animal motivation further impact the data.^18^ In addition to correlations with muscle mass, in our preclinical studies, we also used muscle contractility as a non‐behavioural, proxy measure of muscle function. We previously showed that these measures are robust indicators of neuromuscular function in aged mouse models.^20^, ^27^ Muscle contractility showed correlations with all five testing methods, further supporting physiological validity of grip testing in mice.

In summary, grip strength is a standard method for assessing muscle function in preclinical and clinical studies. This work informs technique, study design, and implementation of preclinical aging studies. It also provides insight into the construct validity of grip strength testing in rodents having reasonable overlap with the conceptual elements of grip strength testing in humans. Our findings suggest a slight edge to the all limb over the bilateral hindlimb method in retest reliability and statistical significance between young and aged mice. Given the clinical significance of grip strength as an indicator of overall health outcomes in older adults, this optimization of grip strength testing techniques in preclinical studies is crucial to the validity and translatability of future preclinical studies exploring the mechanisms of sarcopenia and potential therapeutics to combat aged‐related decline in motor function.

## Funding

This work was supported by funding from NIA/NIH R56AG055795 and R03AG067387 to WDA, R01AG067758 to WDA and BCC, and R01AG044424 to BCC.

## Conflict of interest statement

The authors declare no conflict of interest.
